# Modified Meta Heuristic BAT with ML Classifiers for Detection of Autism Spectrum Disorder

**DOI:** 10.3390/biom14010048

**Published:** 2023-12-29

**Authors:** Mohemmed Sha, Abdullah Alqahtani, Shtwai Alsubai, Ashit Kumar Dutta

**Affiliations:** 1 Department of Software Engineering, College of Computer Engineering and Sciences, Prince Sattam Bin Abdulaziz University, Al-Kharj 16278, Saudi Arabia; aq.alqahtani@psau.edu.sa; 2 Department of Computer Science, College of Computer Engineering and Sciences, Prince Sattam Bin Abdulaziz University, Al-Kharj 16278, Saudi Arabia; sa.alsubai@psau.edu.sa; 3 Department of Computer Science and Information Systems, College of Applied Sciences, Almaarefa University, Riyadh 11597, Saudi Arabia; adotta@mcst.edu.sa

**Keywords:** autism spectrum disorder, autism screening, bat algorithm, artificial neural networks, decision tree, k-nearest neighbours

## Abstract

ASD (autism spectrum disorder) is a complex developmental and neurological disorder that impacts the social life of the affected person by disturbing their capability for interaction and communication. As it is a behavioural disorder, early treatment will improve the quality of life of ASD patients. Traditional screening is carried out with behavioural assessment through trained physicians, which is expensive and time-consuming. To resolve the issue, several conventional methods strive to achieve an effective ASD identification system, but are limited by handling large data sets, accuracy, and speed. Therefore, the proposed identification system employed the MBA (modified bat) algorithm based on ANN (artificial neural networks), modified ANN (modified artificial neural networks), DT (decision tree), and KNN (k-nearest neighbours) for the classification of ASD in children and adolescents. A BA (bat algorithm) is utilised for the automatic zooming capability, which improves the system’s efficacy by excellently finding the solutions in the identification system. Conversely, BA is effective in the identification, it still has certain drawbacks like speed, accuracy, and falls into local extremum. Therefore, the proposed identification system modifies the BA optimisation with random perturbation of trends and optimal orientation. The dataset utilised in the respective model is the Q-chat-10 dataset. This dataset contains data of four stages of age groups such as toddlers, children, adolescents, and adults. To analyse the quality of the dataset, dataset evaluation mechanism, such as the Chi-Squared Statistic and *p*-value, are used in the respective research. The evaluation signifies the relation of the dataset with respect to the proposed model. Further, the performance of the proposed detection system is examined with certain performance metrics to calculate its efficiency. The outcome revealed that the modified ANN classifier model attained an accuracy of 1.00, ensuring improved performance when compared with other state-of-the-art methods. Thus, the proposed model was intended to assist physicians and researchers in enhancing the diagnosis of ASD to improve the standard of life of ASD patients.

## 1. Introduction

ASD is a lifetime neurodevelopmental disorder [1,2,3] that affects brain activity by damaging the nervous system. People with ASD struggle to manage social life due to repetitive behaviour, restricted interests, and reactivity to sensory stimuli [4,5]. Generally, it is categorised into three types, such as Asperger’s syndrome, childhood disintegrative disorder, and pervasive developmental disorder. It is mainly caused by genetic and environmental factors. Additionally, it comes up with co-occurring conditions, like bipolar disorder, ADHD (attention deficit hyperactivity disorder), depression, sensory processing disorder, disruptive behaviour disorder, etc. As it is usually identified in early childhood [6,7], early identification is needed for the primary treatment to avoid consequences and to improve the quality of life of ASD patients. Generally, traditional screening of ASD is carried out by trained professionals with the assessment of observing the behaviour of the patients. It is a time-consuming and expensive process that leads to delays in treatment. Automation in ASD identification is needed to improve the efficacy of ASD detection. Recently, technologies like AI (artificial intelligence) have been utilised to automate the screening process in every group of people [8,9]. ML algorithms can handle multidimensional and complex data, capturing intricate patterns and relationships that may not be apparent through conventional clinical evaluations. This is particularly beneficial in dealing with the intricate and diverse nature of ASD symptoms. They can identify subtle patterns and correlations within extensive datasets, providing a more data-driven and objective perspective on ASD diagnosis. ML models apply predefined algorithms consistently, providing an objective and quantifiable analysis of various features and indicators associated with ASD. This contributes to a more standardized and reliable diagnostic process. Thus, a ML approach complements traditional clinical methods by offering a data-driven, objective, and comprehensive analysis of ASD, leading to more accurate and timely diagnoses. Therefore, the integration of ML into clinical practice has the potential to revolutionize ASD diagnosis and contribute to improved patient outcomes.

Correspondingly, several conventional methods have strived to achieve better ASD identification systems, like ML and DL [10,11]. Diverse symptoms detected in ASD patients are taken as the features of the ASD detection system. Additionally, medical imaging, EEG reports, sensor devices, and text data from the assessment are considered as the features. Some existing techniques considered eye tracking data and behavioural assessment ASD identification features. The widely utilised ML classifiers are CNN (convolutional neural network), AB (AdaBoost), RF (random forest), DT (decision tree), KNN (k- nearest neighbour), NB (naïve bayes), LR (logistic regression), SVM (support vector machine), and LDA (linear discriminant analysis). In this existing system, the DL technique has been utilised for ASD detection. It has been accomplished by using 1D CNN algorithm through the ASD dataset. This dataset comprises adult, children, and adolescent data. The results show the better performance of this existing identification system. On the contrary, several ML classifiers are utilised in ASD identification. The features of this existing system are taken by computing the head RR (rotation range) and ARPM (amount of rotation per minute) in the pitch, yaw, and roll of the ASD and TD (typical development) children. The classifiers utilised in this traditional method are SVM, LDA, DT, RF, and ENS. From the analysis outcome, it is concluded that the DT classifier attained a higher accuracy of 92.11% [12]. Correspondingly, the DL based model has been utilised to detect ASD in children. MobileNet with dense layers of two has been used for feature extraction and classification. The data of 3014 images comprised children with ASD and children without ASD. The outcome of this existing model states the better performance of the model with 94.6% accuracy [13]. Several conventional techniques attained ASD identification with satisfactory accuracy in toddlers, children, adolescents, and adults but lacked accuracy and speed in managing huge data in the identification network. 

To resolve the issue, the projected system employs MBA with ANN, modified ANN, DT, and KNN to identify ASD. The proposed study on ASD identification is motivated by the limitations in present approaches. While conventional techniques have shown satisfactory accuracy, they find challenges in identifying ASD accurately in specific age groups or diverse demographics. As the existing studies have not fully addressed the complexities associated with ASD, such as its varied manifestations and the need for early detection, the major complication lies in achieving high accuracy across different scenarios. Therefore, this study aims to fill these gaps by introducing a novel approach that specifically addresses the limitations of current methods. By doing so, it strives to contribute significantly to the field of ASD identification, providing a more accurate, nuanced, and timely solution to better meet the diverse needs of individuals affected by ASD. The Q-chat-10 dataset has been utilised in the respective system to identify ASD in toddlers, children, adolescents, and adults. It is collected from the questionnaire called Q chat-10 comprises 10 questions. The model’s features are taken from the answers obtained from the Qchat-10. The dataset is evaluated using the chi-squared statistic and *p*-value technique to analyse the eminence of the dataset with the proposed system. In the respective model, the BAT velocity is modified in order to improve the accuracy and computation in ASD identification. At first, the ASD dataset is passed to the pre-processing stage. Here, the label encoding method is used to convert the data from categorical columns to the data of numerical. Also, data normalisation is used to normalize the features of data. Formerly, the data is distributed into data of training and data of testing. The data fed for training goes over the classification process by the combination of MBA with ANN, modified ANN, DT, and KNN systems. After classification, the data pass through the prediction phase and testing model, where the model is evaluated with the training data. Finally, the respective identification model is examined with metrics of performance to calculate the efficacy of the proposed identification system. 

The major contribution of the projected system is as follows:

- To utilise dataset evaluation techniques, such as the chi-squared statistic and *p*-value technique for evaluating the distinction of the Q-chat-10 dataset with the respective research.
- To employ MBA with ANN, modified ANN, DT, and KNN to improve the speed and accuracy of ASD identification.
- To optimise the performance of the MBA, velocity is adjusted to improve the efficacy of the proposed system.
- To compare the performance of MBA-based ML classifiers with BAT-based ML classifiers to examine the effectiveness of proposed ASD identification.
- To calculate the performance of the proposed system with performance metrics such as precision, accuracy, F1-score, recall, confusion matrix, and roc curve.

### 1.1. Motivation of the Proposed Research

The ASD severity depends on the social life of the patients, such as disablement in communication and repetitive and constrained patterns in their behaviour. Presently, there is no medication available to treat ASD. However, particular medicine can support with the associated symptoms such as insomnia, seizures, depression, etc., correspondingly, to evade the consequences of the ASD, it is necessary to identify the disorder in the primary stage. To attain this, several conventional research projects have endeavoured to achieve effective ASD prediction. Nevertheless, limited by accuracy, computation and handling larger datasets. With the motive of attaining enhanced ASD prediction, proposed method utilised MBA with the ML algorithms such as ANN, modified ANN, DT, and KNN for the ASD and non-ASD classification through the Q chat-10 dataset. 

### 1.2. Significance and Scope of the Respective Model 

The proposed model utilised the advantages of MBA with ANN, modified ANN, DT, and KNN to accomplish ASD and non-ASD classification. The respective classification supports in identifying the ASD in the early stage of the children. The initial ASD identification helps in attaining primary ASD treatment to diminish the future consequence of the disorder. Moreover, the proposed model intended to assist the physicians to plan the treatment by providing enhanced ASD identification system. The early ASD identification is necessary because primary diagnosis enhances the functional outcomes and improve the quality of life. Since ASD is a lifelong disability, the early prediction help the patients and family members to manage the symptoms. Additionally, it supports in minimising the risk of future life issues like school, college, or sustaining employment and managing mental health, etc. 

### 1.3. Paper Organisation

This paper is categorised based on effective methods for identifying ASD, whereas analysis of the existing works is performed on a similar domain with a different approach and shown in Section 2. Further, Section 3 signifies the methodology executed in the proposed system. The results and outcomes attained by the proposed method are shown in Section 4. Lastly, the conclusion and future work of the proposed model is determined in Section 5.

## 2. Review of Literature

Several conventional technologies involved in the screening of ASD in toddlers, children, adolescents and adults are discussed in this section. Further, the complications recognised in the traditional system are also discussed. ASD is a multifaceted developmental and neurological disorder categorised based on the characteristics in the growth of social skills like communication, intellectual capabilities, and constrained monotonous behaviours [14,15,16]. Since it is a behavioural disorder, early diagnosis will help the patients to improve their quality of life. An effective screening system is needed for primary treatment [17,18]. For this purpose, this traditional system utilised an ML-based system for ASD identification. Several classifiers, such as SVM [19,20], LR, KNN, CNN, and NB, have been analysed in the existing system. A non-clinically ASD dataset, which comprises adolescents, children, and adult data, has been utilised for the identification. The outcome of this existing system shows that CNN has accomplished better performance [21].

Similarly, the ML model [22,23,24] has been utilised for the screening of ASD [25]. For that, the CNN algorithm has been utilised. The data has been extracted with the application on the mobile device. The identification system has been further compared with other algorithms to calculate the efficacy of this traditional deduction system. From the outcome, it has been revealed that this existing system accomplished better performance in ASD detection [26]. Accordingly, the ML technique has been utilised for ASD detection. This traditional system has employed diverse feature-scaling techniques through feature-scaled datasets. Eight classifiers have been utilised in this existing technique, such as AB, RF, DT, KNN, GNB, LR, SVM, and LDA. The dataset comprises toddlers, children, adults, and adolescent’s data. The experimental outcome shows that the AB achieved better accuracy in detecting ASD [27]. Similarly, in the same way, an ML [28,29] based system has been utilised for ASD [30,31] detection. For this, various classifiers have been analysed, such as MLP, SMO, LR, SL, LB, ICO, RAB, and LMT. Four types of data collected from toddlers, children, adolescents, and adults has been utilised in this existing method. The outcome of this research shows that the MLP classifier and feature selection with Relief F has produced better performance [32]. 

In the same way, the ML model with OS (owl search) algorithm has been utilised for detecting and classifying ASD. Here, the normalisation process of min-max has been utilised in the pre-processing stage. Additionally, the selection of features is performed through the OS algorithm to derive subsets of features and the BSAS (beetle swarm antenna search) algorithm with ID3 (iterative dichotomiser 3) has been utilised for classification. Benchmark dataset has been utilised in the classification. From the end results, it has been concluded that the traditional system accomplished a better performance [33]. Likewise, diverse ML classifiers have been utilised to detect ASD through the ASD dataset of toddler, child, adolescent and adult data. From the end outcome, it is signified that the SVM model attained better accuracy with 97.82% than the other classifies [34]. Correspondingly, the ML system has been utilised for the identification of ASD. Several models have been utilised, such as SVM, KNN, AdaBoost, and NB, in this identification system. The ASD dataset has been utilised in this traditional system. This dataset consists of 16 attributes of 703 ASD-affected people and non-affected people. This analysis shows that the SVM, NB has produced a better performance with less error rate [35]. In this conventional technique, an ML algorithm has been utilised to detect ASD in adults. Here, a behavioural diagnostic tool called ADOS (autism diagnostic observation schedule) has been utilised to extract the five behavioural features for the detection model. It has been examined with the ADOS module 4 in 673 samples of clinical data of 385 adults. This dataset comprises both adults with and without ASD. The model outcome accomplished better performance in detecting ASD [36]. 

In the same way, AC (association classification) has been utilised in the ASD classification. Seven diverse algorithms have been analysed in the traditional system to detect correlations among the features in the detection of ASD. From the comparison result, it has been found that the WCBA algorithm attained better accuracy of 97% [37]. Accordingly, the ML system has been utilised for the detection of ASD in toddlers through the Q-CHAT (quantitative checklist for autism in toddlers) dataset. For this, five diverse algorithms has been utilised, such as RF, NB, SVM, LR, and KNN. From the analysis, it has been concluded that the SVM classifier accomplished higher accuracy of 95% in ASD identification [38]. Likewise, the ML-based model has been utilised in ASD identification. FANN (feed-forward artificial neural network) has been utilised with M-CHAT-R (modified checklist for autism in toddlers, revised). From the end outcome, it has been concluded that the existing model accomplished a better performance [39]. In the same way, four ML algorithms have been utilised to classify ASD and TD (typical development). For that, SVM, DT, RF and LDA has been utilised in the existing model. Here, eye-tracking data has been used as the input data. The outcome of this model signifies the SVM classifier has attained a better level of accuracy with 92.31% [40]. 

Likewise, ML-based classification has been utilised in ASD identification in the traditional system. For that, several classifiers have been utilised, such as DT, RF, NB, KNN, SVM [41], LR, AdaBoost, neural network, and MLP has been utilised. The input data has been acquired from the ASD dataset. From the analysation result, Adaboost, KNN, neural network and DT produced better results with a higher accuracy value of 0.9995, 0.9834, 0.9925, and 0.9786 [42]. Similarly, ML-based five classifiers have been analysed in the traditional system of ASD identification. The system’s features have been taken from evaluation, behavioural and kinematic and neuroimaging data. SVM, LDA, DT, RF and KNN have been utilised as the classifiers. From this outcome, it has been concluded that the KNN has produced a better performance with a higher accuracy of 88.37% than other classifiers [43]. Correspondingly, an ML-based ASD identification system has been utilised in the existing model. Here, the CNN algorithm has been used to detect ASD by imaging brain data called ABIDE (autism brain imaging exchange) dataset. The existing model intended to classify ASD and manage focus on patterns in connectivity of function. The result of this traditional system signifies better performance in ASD identification with an accuracy of 70.22% [44]. 

### Problem Identification

This section signifies the limitations and challenges identified in the conventional researches on the ASD classification.

- The ML-based CNN system was utilised to identify ASD, but lacks in handling larger datasets and speed [23].
- The ML-based system was analysed for the detection of ASD, but could not handle larger datasets [32].
- ML system was utilised for ASD identification, but the data quantity was insufficient for all stages of people [27].

## 3. Proposed Methodology

Autism is a neurological and developmental disorder that affects neural function in the brain. The person with ASD finds difficulties in social communication, understanding, and responding to the people. Autism is diagnosed on the basis of two core behaviour patterns, such as repetitive behaviour and restricted interests. Additionally, complications in social life, such as communication and interaction, are caused due to particular alterations in diverse areas in the brain and the connection with one another. The parts of the brain affected by autism are the cerebral cortex, amygdala, hippocampus, brain stem, basal ganglia, corpus callosum, and cerebellum. The following table signifies the particular regions of the brain affected by ASD. 

Table 1 signifies the part of brain affected by ASD and their chief functions in the human behaviour. ASD disturbs the significant areas in the brain and cause several challenges such as restricted behaviours, reciprocal social communication, body movements, etc. Correspondingly, ASD is characterised into three main stages such as level 1, level 2 and level 3. The precise description of the levels are represented in the following:

- **ASD Level 1**

It is the highest functioning and mildest form of autism. People with level 1 ASD can successfully speak in full complete sentences. However, it is hard to engage in complete back and forth communication. People with level 1 ASD have challenges in planning, organisation, switching between tasks, etc.

- **ASD Level 2**

ASD level 2 affected persons have challenges in social communication. Children with ASD level 2 find difficulty in verbal and non-verbal communication.

- **ASD Level 3**

It is characterized in several challenges in social life such as inflexible behaviour, difficulty in handling changes in routine, restrictive and repetitive behaviour. 

**Figure 1:**
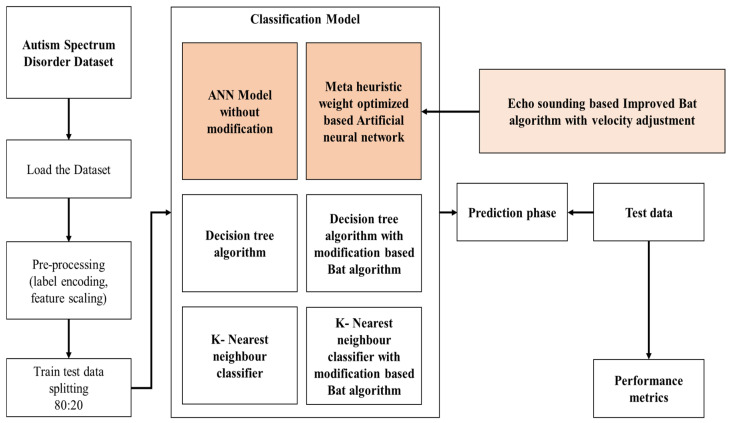
Design Flow of the Projected System.

Correspondingly, people with ASD can live a normal life with the correct assistance and resources. To attain this, early detection of ASD is needed to receive precise treatment of the disorder. Moreover, primary intervention, community support and education is needed to support people with ASD to accomplish their goals and leads a satisfying life. For this purpose, conventional researches used the ML technology for the ASD identification which have attained satisfactory results but lacked in speed and accuracy. Therefore, the projected system utilised ANN, modified ANN, DT, and KNN with MBA to improve the performance of ASD identification. Here, the velocity of the BA is modified to enhance the speed of convergence and accuracy of the ASD identification. Further, the projected system efficacy is examined with metrics to evaluate the performance of the proposed research. Figure 1 represents the design flow of the projected system.

Figure 1 depicts the proposed research comprises of data selection, data evaluation pre-processing, data splitting, classification and prediction phase. The process in the proposed research is depicted in the following sections:

### 3.1. Data Selection

The projected model aimed to identify autism in children and adolescence through the Q-chat-10 dataset. This dataset comprises 20 features defining significant autistic characters. The data is acquired from the assessment with the questionnaire comprising 10 questions taken by the family member of contributing children and adolescents. It is intended to analyse the identification characteristics of autism and to enhance the ASD classification. It is a non-matrix format dataset containing four types of data: sequential, univariate, multivariate, domain-theory, text, and time-series. The dataset is intended to be applicable for classification purposes. The attribute type is categorized into binary, categorical, and continuous types. The dataset is acquired from the following link: https://github.com/raj-shr-git/Autism_Spectrum_Disorder/blob/master/Autism_Child_data_in_excel.xlsx (accessed on 26 December 2023). Table 2 signifies the attributes of the dataset. 

Table 2 signifies the characters, kind, and specifications in the dataset assessment. It is acquired from the autism test screening app. The screening score is the final result acquired from the assessment of the dataset. Correspondingly, the value of instances in the dataset is 292, and the value of attributes of the dataset is 21. Additionally, the dataset features comprises 100 base questions which is called the Q-chat-10, where the possible answers are categorised as always, sometimes, usually, never, and rarely. Consistently, the values of the items are represented as “0” or “1”. Table 3 signifies the questions given in the assessment of ASD screening for children dataset.

Table 3 represents the questions from the ASD assessment in the Q-chat-10 dataset. It comprises two age groups data such as toddlers and adolescents. In the assessment, the responses to the questions from 1 to 9 is never, rarely, or sometimes, then the value 1 is allocated to the question. Concurrently, if the tenth question response is usually, sometimes, or always, then 1 is allocated to that question. Primarily, in the proposed research, the ASD dataset is loaded in the respective system. 

### 3.2. Dataset Evaluation 

In the proposed system, the eminence of the dataset is evaluated with the significant examination called the chi-squared test and *p*-value.

- **Chi-Squared Statistic**

It is the statistical hypothesis test that is utilised to investigate the contingency tables in the case of large sample size in the dataset. Particularly, it analyses whether the two categorical variables are influencing the statistics of the test. Correspondingly, test statistics are the calculation of the difference among the observed and expected frequencies in the contingency table. It measures the degree to check whether the utilised data diverges and has no similarities among the tested variables.

- ***p*-value**

*p*-value is the statistical calculation that are utilised to validate a hypothesis against the utilised data. It measures the probability with the assumption of null hypothesis, of acquiring an outcome that are equal to extravagant than the observed outcome. Consequently, it is the calculation of the evidence in contradiction of the null hypothesis. Accordingly, a low *p*-value specifies that there is a strong evidence for eliminating the null hypothesis and recommends the important dependence among the variables in the data that are being tested in the method. 

### 3.3. Pre-Processing 

Predominantly, pre-processing is used to formulate the data for the classifications. It augments the quality of the data for generating it suitable for the precise classification procedure. The ASD dataset is fed to the pre-processing, where the label encoding technique is utilised to transform the data from categorical columns into numerical data. Here, data normalisation, also known as feature scaling, is used to normalise data features. Data normalisation is also known as feature scaling, was used as part of the pre-processing step. Feature scaling is a technique used to standardise or normalise the range of independent variables or features of the dataset. The main objective is to ensure that all features contribute equally to the computation and prevent some features from having a disproportionate impact on the learning algorithm. The normalisation process involves scaling the features so that they have a mean of 0 and a standard deviation of 1. This is achieved by using the technique min-max scaling approach.

### 3.4. Data Splitting

The data splitting is used by the ML based researchers to evade the over fitting of data in the model. Moreover, splitting of data assists the system to evaluate the performance of the ML algorithms. In the proposed research, data is divided into data of training and data of testing. The training set of data is utilised to train the model for learning the hidden patterns in the data. Accordingly, data of testing is used to examine the model after the training process. Consequently, training set is used for the classification process. 

### 3.5. Classification

In the classification process, the respective approach used the MBA with three classifiers such as ANN, modified ANN, DT, and KNN, to improve the classification efficacy. Here, the BA is modified with velocity adjustment in the respective system. The model utilises nonlinear variation to enhance the velocity in the diverse BAT population. The local search capacity is enhanced by the technique of optimal value modesty. Lastly, minimising the perturbation in every BAT individual is efficient in position for the local extremum. This MBA optimisation combines effective classifiers like ANN, modified ANN, DT and KNN to improve the accuracy and convergence speed. Finally, the projected model is examined in the prediction phase to evaluate the identification efficacy. The proposed algorithm and the internally compared classical algorithm is depicted in the following sections. 

#### 3.5.1. BA (Bat Algorithm)

It is the simple BA that signifies the solution of feasible to the space of solution. The individual optimal is set by comparing values of a unique function for every BAT for diverse fitness functions. Formerly, the process of BA starts with the calculation of velocity, emission of pulse rate, wave frequency, and every BAT loudness in the population. Then, the iteration proceeded, and the solution of optimal is produced. Finally, a global optimum solution is identified in the system. 

The velocity, position and velocity is signified in the following Equation (1)

(1) $${freq}_{i}={freq}_{min}+{(freq}_{max}-{freq}_{min})\beta$$

(2) $${sp}_{k}^{t}={sp}_{k}^{t-1}+\left(pos_{k}^{t-1}-pos_{*}\right)freq_{K}$$

(3) $$pos_{k}^{t}=pos_{k}^{t-1}+{sp}_{k}^{t}$$

Here, ${freq}_{i}$ represents the frequency of emitted waves, where ${freq}_{max}$ and ${freq}_{min}$ signifies the frequency of maximum and minimum. The parameter in the frequency of maximum and frequency of minimum is Fmax: 2 and Fmin: 0. Moreover, parameter of K is 5 in the respective model. β is the generated number. The main purpose of β is to introduce a random element into the calculation of the frequency ${freq}_{i}$ of emitted waves. This randomness contributes to the exploration of the search space during the optimization process. In the BA, the frequency of emitted waves is a crucial parameter that affects the bat’s movement. The equation indicates that the frequency is calculated based on a range defined by ${(freq}_{max}-{freq}_{min})$ beta determines where within this range the frequency will fall. ${sp}_{k}^{t}$ and $pos_{k}^{t}$ represent the speed as well as position. pos signifies the current optimal position of the system. The updated local search position is represented in Equation (4). It is calculated by taking the average of the entire loudness of the bat. The iteration is inversely proportional to the bat’s loudness. The parameter of the alpha and gamma are 0.9 and 0.9, respectively.

(4) $${pos}_{new}=pos_{*}+\varepsilon A^{t}$$

where, the pulse emission rate increase, where the parameter of A is 2. Then,

(5) $${lud}_{k}^{t+1}=\propto lud_{k}^{t}$$

(6) $$r_{k}^{t+1}=r_{k}^{0}[1-\exp\left(-\gamma t)\right]$$

Here, the value of ∝ as well as γ is unchangeable, where the parameter of r is 1, alpha is 0.9 and gamma is 0.9. The primary pulse emission rate $d_{k}^{t}\to 0,r_{k}^{t}\to r_{k}^{0},t\to \infty$ is based on any γ>0 and 0 < ∝ < 1. Thus, the parameters such as ∝, β and γ tend to control the dynamics, namely, loudness decay rate, convergence rate, and pulse emission rate of the algorithm. When these parameters are adjusted, this allows the fine-tuning of the behaviour of the modified bat algorithm based on the characteristics of the optimisation problem. By performing optimisation, the algorithm is improved in terms of convergence acceleration, balanced exploration-exploitation, diversity maintenance, robustness, and enhanced solution quality. 

#### 3.5.2. MBA (Modified Bat Algorithm)

**Figure 2:**
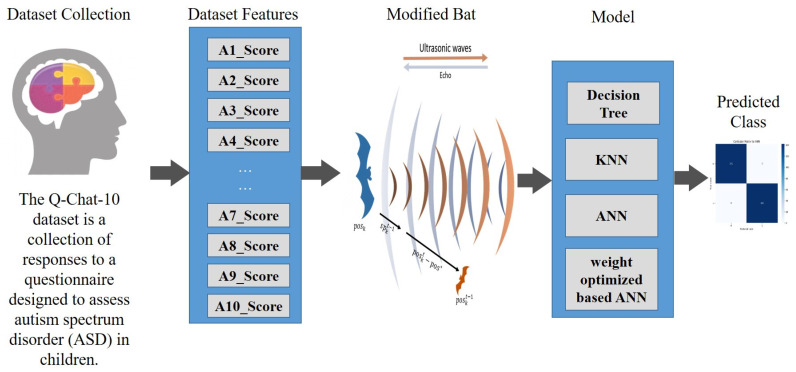
Identification Mechanism in MBA with ANN-DT-KNN.

The projected system modifies the BA to improve the speed of convergence and accuracy of the identification system. For that, the model utilised the random perturbation of trend and optimal orientation in the BA. The identification process of the MBA is depicted in Figure 2.

Figure 2 represents the ASD identification of the projected system where the MBA is utilised with other three ML classifiers like ANN, modified ANN, DT, and KNN. Consequently, Equations (1) and (3), signifies the velocity of the step size of the upgraded position of the bat individual. Henceforth, Equation (2) represents the global searching capability. The projected system utilised a non-linear factor in Equation (2) to improve the capability. The speed upgrade is signified in Equation (7) where L=2a∗b−a expands the bat population. Here, a denotes the convergence, and b denotes the random number.

(7) $$sp_{k}^{t}={sp}_{k}^{t-1}+L*freq_{k}$$

Then, the convergence is represented in Equation (8).

(8) $$a=2-{\left(\frac{t}{T_{max}}\right)}^{2}*(e-1)$$

Here, e is the natural constant, and $T_{max}$ is the higher iteration value. It is found by calculating the distance in the local search stage. The factor of convergence reduced with the iteration value. Then, the factor of convergence’s degree decay is light in the iteration’s initial stage so that the larger L value effectively moves in the larger to identify the solution of global optimal. The L value will be smaller, and the degree of decay will be increased in the iteration’s final stage. Here, the randomness increases the difference in the nonlinear trend of decline so that the growth and searching capability are balanced in the identification system. The position update in the MBA is represented in Equation (9)

(9) $${pos}_{new}={pos}_{*}+D_{k}\cdot e^{l-1}\cdot \sin(\pi l)$$

Here, the distance among the BAT previously the update of location and optimal individual in current is represented by $D_{k}=|pos_{k}^{t-1}-C\cdot {pos}_{*}$.

Where the c=2.b is the factor that impacts the distance among the BAT individual $pos_{k}^{t-1}$. Here, b represents the random number where e is the constant and l signifies the value produced in a certain range of [−1, 1]. If C < 1, the influence degree decreases, and if C > 1, the influence degree increases. The BAT individual is managed by sin⁡(πl) where the bat individual is farer in the system to the current optimal individual. 

In the projected identification system, convergence accuracy is enhanced, and the local extremum is avoided by utilising the random perturbation method. Hence, the perturbation mechanism is utilised in the MBA to improve the accuracy of the respective identification model, represented in Equations (10) and (11), where $pos_{k}^{\prime }$ represents the position of the k th bat individual.

(10) $$per=per_{max}-\left(per_{max}-per_{min}\right)\cdot \cos(\frac{\pi }{2}\cdot \left(\frac{1}{T_{max}}-1\right))$$

From Equation (10), the permutation coefficient decreases with the increase in iteration.

(11) $$pos_{k}^{\prime }=pos_{k}+per\cdot \mu \cdot (pos_{k}-pos_{rand})$$

The increase in iteration makes the position $pos_{k}^{\prime }$ decrease in the respective system, which increases the population diverts with the capacity to evade local extremum. Hence, in Equation (10), the coefficient of the perturbation decreases with iteration times to make the disturbance in the position in Equation (11). This method enhances population diversity and reduces the chances of getting into the local extremum. The capability of fine mining is managed in the optimal solution in the iteration’s final stage. This MBA was utilised to improve the convergence speed and accuracy of the projected ASD identification. Algorithm 1 signifies the classification process in MBA. The selected features of the model are 10, 13, and 17.
**Algorithm 1:** Modified Bat Algorithm1. Set the function of the objective f(pos) as well as individulas of bat population. 2. Denoteloudness (lud), velocity (sp),  pulse emission rate (r),  frequency (freq_max_, freq_min_), iteration of higher value (T_max_), and dimension (D). 3. Calculate function of fitness for every individual  and denote the value of current optimum (pos_*_) as well as position (pos). 4. Generate the iteration in the counter t to 1. 5. While t is less than or equal to T_max_, generate the steps given below:  a. Upgrade rate of pulse emission, velocity,  as well as position of every bat through Equations (1), (3), and   (7) Then, to take in position (pos_t_).  b. Calculate the fitness valuein position (pos_t_).  c. Create a similar random value p.  d. If p is less than the rate of pulse emission (r),  do a local search nearby the current optimal   individual to produce a new position (pos_new_).  Restore the value of pos_t_ with pos_new_.  e. Perturb the position (pos_t_) through Equations (10)  and (11) to make a new position (pos_0_).  f. Calculate the function of fitness for the new position (pos_0_).  g. If the value of fitness of pos_0_ is better than  the value of fitness of pos_t_, restore pos_t_ with pos_0_.  h. Generate a uniform random number rand.  i. If rand is greater than the rate of pulse emission (r)  multiplied by the value of fitness of pos_t_ minus   the value of fitness of the prior iteration’s best individual (pos_t−1_), accept   the upgraded pos_t_ as the position of current individual.  j. Upgrade the loudness (lud) and rate of pulse emission (r)  through Equations (5) and (6), respectively.  k. If the value of fitness of pos_t_ is better than the value of fitness  of the solution of current optimal (pos_*_),   ugrade pos_*_ and set it as the current best individual.  l. Increase the iteration counter (t). 6. Output the solution of global optimal (pos_*_).

#### 3.5.3. ANN Algorithm

ANN is the ML-based algorithm widely utilised in recognition of images, identification of speech and processing of natural language. It has the capability of handling non-linear and complex data. The architecture of ANN comprises three layers such as input layer, layers of hidden and layers of output. The complexity is altered by removing and adding of the neurons. Initially, the available data is divided into subsets of three. Where the subsets are utilised for testing, training, and validation. Table 4 shows the summary of the ANN approach.

The classification performance is evaluated in the validation subsets. The main advantages of ANN is non-linearity, adaptability to learn the new data, better generation of patterns even through the noise data, and enhanced accuracy in the model. Algorithm 2 signifies the classification process in ANN.
**Algorithm 2:** Artificial Neural Networks**Step 1:** Start fix the weights (w) to random values. **Step 2:** Propagation Farward Pass the input neuron values (x) through hidden neurons to the output neuron. Evaluate the output neuron values (ANN probabilities). Calculate the error function using Equation (A1). **Step 3:** Propagation Backward Evaluate the gradient (g) for the network neurons. Adjust the weights (w) using the gradient. Increment the iteration count (p) by one. Repeat steps 2 and 3 until the chosen error criterion is met. **Step 4:** Iterate (recommended) Repeat the entire process starting from step 1 to minimize the likelihood of obtaining a sub-optimal ANN.

#### 3.5.4. Weight Optimisation Based ANN

The weight optimised based ANN is the technique that trains the functional link neural network using the behaviour instances of the past network and use weight based optimisation for optimising the parameters of the functional model. Accordingly, weight optimised based ANN is the least complex than the traditional ANN that are accomplished of complex input-output mapping and better non-linear identification. Table 5 shows the hyperparameters with corresponding values for the modified ANN approach.

The functional link used in the technique utilises activation function which has the efficient gradient and least probability of early saturation in the system. Moreover, features assist in the enhanced speed in training of network for the autism detection in the respective research. Similarly, effective computation supports in identifying finest weight parameter in the system. This computation have exchange diversification and intensification, which enhances the global optimization. Algorithm 3 represents the weight optimised based ANN.
**Algorithm 3:** Weight Optimization Based ANN1. Start n (amount of set of weight).  $IN=\left\{{In}_{1},{In}_{2},\cdot \cdot \cdot ,{In}_{n}\right\},$ where every In_i_ parameter (set of weight) in a system That are initialized as the following: ${In}_{i}=\{{weg}_{i},1,{weg}_{i},2,\cdot \cdot \cdot ,{weg}_{i},n\}$ 2. Compute mean in set of weights (INmean) among the IN. 3. Compute fitness of all In_i_ in IN (Algorithm 2 (Fitness process)) and choose xi with utmost fitness as best solution (INteacher). 4. Execute INnext (next population) through IN, INmean, INteacher and TF  for i = 1 to n (amount of In_i_ in IN)TF = round(1 + rand(0, 1)(2 − 1))   In_i_ = In_i_ + rand(1)(In_teacher_ − T_F_ × IN_mean_)   IN_next_ (i) = In_i_  endfor 5. Upgrade IN by contrasting fitness of solutions (IN_i_) in IN and IN_next_:        for i = 1 to n (amount of In_i_ in IN)        $if(fitness(IN(i)<fitness({IN}_{next}(i)))$         $IN\left(i\right)={IN}_{next}\left(i\right)$        endif        endfor 6. Randomly choose two solutions
(In_i_ and In_j_) from X and improve:  for k = 1 to n (amount of In_i_ in IN) Randomly select In_i_ and In_j_ from IN Get fitness of In_i_ as F_i_ $=Fitness\left(\varphi ,{In}_{i},t,µ\right)\left(Algorithm\;2\right)Get\;fitness\;of\;{In}_{j}\;as\;F_{j}$ $=Fitness\left(\varphi ,{In}_{j},t,µ\right)\left(Algorithm\;2\right)$    $if\left(F_{i}<F_{j}\right)$      ${IN}_{new}\left(i\right)={In}_{i}+rand\left(1\right)\left({In}_{j}-{In}_{i}\right)$    $Else{IN}_{new}\left(j\right)=Inj+rand\left(1\right)\left({In}_{i}-{In}_{j}\right)$    ifend  endfor 7. Keep Elite Solution (In_i_ with greater fitness):   for i = 1 to n (amount of In_i_ in IN)    Compute fitness of each In_i_ in IN as      $F_{i}=Fitness\left(\varphi ,{In}_{i},t,µ\right)\left(Algorithm\;2\right)$  endfor Choose one In_i_ with maximum F_i_ as IN_elite_, which serves as elite set of weight in IN 8. Identify weak set of weights (Set of weights with greater fitness) and replace with IN_elite_. 9. Alter duplicate set of weights by mutation operation on duplicated set of weights   For I = 1 to n (amount of In_I_ in IN)       $if\left(i\neq {mIn}_{i}\;and\;F\left(i,1\right)==mIn\right)$       $mIn\;and\;{mIn}_{i}\;are\;greater\;fitness\;value\;and\;its\;index,\;respectively$   $for\;k=1:1:round\left(\frac{l}{4}\right),\;l\;is\;thelength\left({In}_{i}\right)$      $j=round\left(mod\left(\left(rand\left(1\right)\times l\right),l\right)\right)$       $minv=\min\left(IN\left(i,j\right)\right)$      $maxv=\max\left(IN\left(i,j\right)\right)$     ${IN}_{new}\left(i,j\right)=\frac{minv+maxv}{2}$     endfor    endif   endfor 10. Analyzing for stopping condition:    $if\left(\begin{array}{c} \mathrm{improvement}\;\mathrm{in}\;\mathrm{fitness}\;\mathrm{of}\;\mathrm{solutions}\;\mathrm{in}\;\mathrm{IN}\;\mathrm{is}\;\mathrm{not}\;\mathrm{significant}\;\mathrm{and}\;\\ \;\mathrm{or}\;\mathrm{greater}\;\mathrm{iteration}\;\mathrm{is}\;\mathrm{reached} \end{array}\right)$    goto Step 11      else        goto Step 2     endIf 11. Exit

#### 3.5.5. DT Algorithm

**Figure 3:**
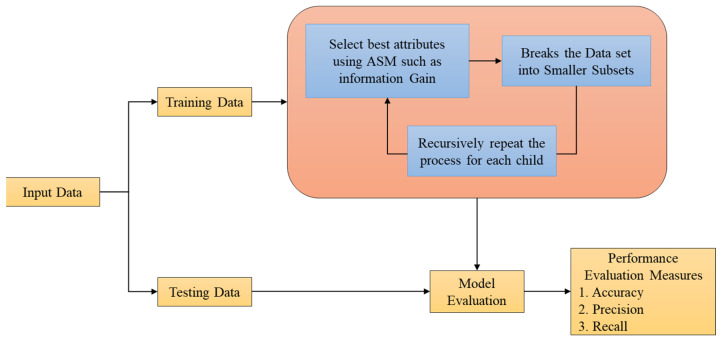
Conceptual Design of DT.

DT is the widely utilised classifier as it is simple in interpretation and data of non-linear visualisation. It is fast, particularly for the analysis of data. It provides higher accuracy, mainly for smaller datasets. The conceptual design of the DT algorithm is signified in Figure 3. 

Figure 3 depicts the architecture of the DT. Generally, it divides data into branches. It selects the best attributes and divides into smaller subsets. Formerly, it produces the tree to enhance the accuracy of the classification. It is mainly categorized into two phases, such as the phase of growth and the phase of the build. In the growth phase, data is divided into small subsets based on better criteria, whereas, in the phase of the build, minimization of branches is done for the production of the tree for an effective gene generalising system. It handles the over-fitting of data and also eliminates the noise nodes. Table 6 shows the hyperparameters with corresponding values for DT approach.

The accuracy of the classification increases in the build phase. Algorithm 4 signifies the classification process in the DT algorithm.
**Algorithm 4:** Decision TreeDecTree(sam, feat) If stopping_condition(feat, feat) = true then a. The. Leaf = createNode( ) b. The leafLabel = classify(sam) c. return leaf root = createNode( ) root. tes_condition = findBestSpilt (sam feat) V = {v|v a possible outcomefroot. test_condition_} For every value vϵV: $a.S_{v}=\left\{s\right|root.\mathrm{test\_condition}\left(s\right)=v\;and\;s\epsilon S\};$ $b.Child=TreeGrowth\left(S_{v},feat\right);$ $c.Add\;child\;as\;the\;descent\;of\;root\;and\;label\;the\;edge\left\{\begin{array}{c} \mathrm{root}\\ \to \;\mathrm{child} \end{array}\right\}\;as\;v$ return root

#### 3.5.6. KNN Algorithm

**Figure 4:**
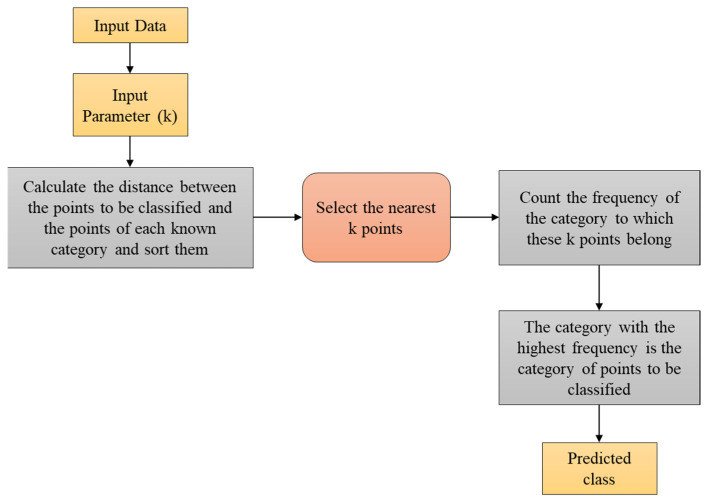
Architecture of KNN Algorithm.

KNN is an effective ML-based algorithm that undertakes the connection among the data as well as available cases. It extracts the new case in the category identical to the available one. It stocks the available data, classifying new data based on similarity. The architectural flow is illustrated in Figure 4. 

Figure 4 illustrates the KNN architecture. It is widely utilised for regression and also classification. It is the non-parametric technique that does not supposition the data. Additionally, it does not learn immediately on the set of training data. As an alternative, it stocks the dataset and accomplishes action in the classification time. It is widely utilised in classification because it has the capability of handling cases of multi-class, easy implementation, and no supposition on fundamental data. Table 7 shows the hyperparameters with corresponding values for KNN approach.

In the KNN process, the distance among the points are computed and the k-nearest points are selected in the system. Further, the frequency is counted and the classification is based on the category with the highest frequency. Algorithm 5 signifies the classification process in the KNN algorithm.
**Algorithm 5:** K-Nearest NeighbourBegin Select the k closest neighbors for every observation I based on the covariates x. Separate the k treated neighbors (w_i_ = 1) and k untreated neighbors   (*I* = 0) for every observation i.  For every observatioI i, do the below steps:  a. Evaluate the average of y_k_ (w = 1) in treated neighbors.  b. Calculate the average of y_k_ (w = 0) in untreated neighbors.  c. Determine the uplift as the difference among the average of   y_k_ (w = 1) and the average of y_k_ (w = 0). End

### 3.6. Prediction Phase

The final phase of the respective research is the prediction phase. The prediction phase examine the performance of the proposed research through the testing set. Correspondingly, the performance of the proposed model is examined using the performance metrics to evaluate the efficiency of the respective system. 

## 4. Results and Discussions

The result attained by the execution of the respective mechanism is presented in this section. Furthermore, the dataset description with the EDA, sample data, performance metrics, experimental results, and comparative analysis of existing techniques are presented.

### 4.1. EDA (Exploratory Data Analysis)

**Figure 5:**
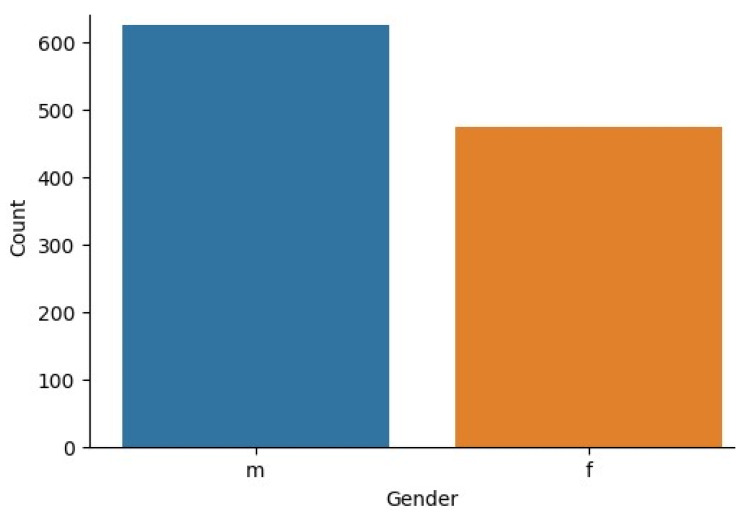
Gender Distribution in Q-Chat-10 Dataset.

The EDA is the analysis method of the utilised dataset to classify general patterns such as features and outliers. Moreover, it used to view and understand the dataset. Correspondingly, the proposed model used Q-chat-10 dataset for the ASD classification. Figure 5 depicts the gender distribution of data in the Q-chat-10 dataset. 

**Figure 6:**
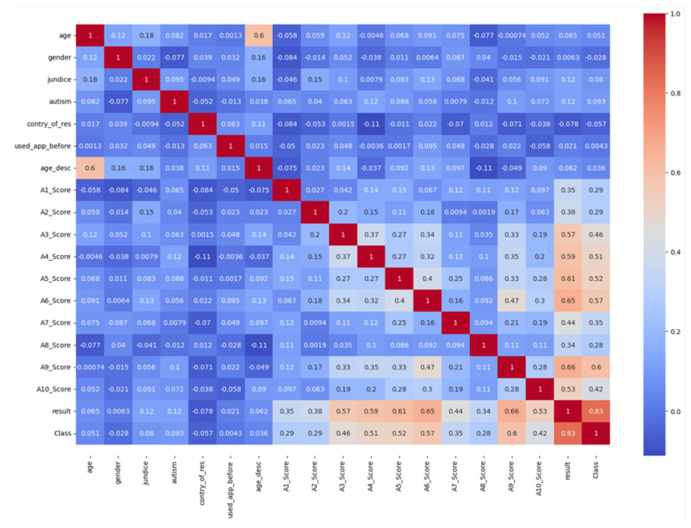
Correlation Map of the Projected System.

Figure 6 represents the distribution of male and female data presented in the used Q-chat-10 dataset. It is identified that the male data is significantly higher compared to the female data. Correspondingly, correlation heat map is illustrated in Figure 6.

**Figure 7:**
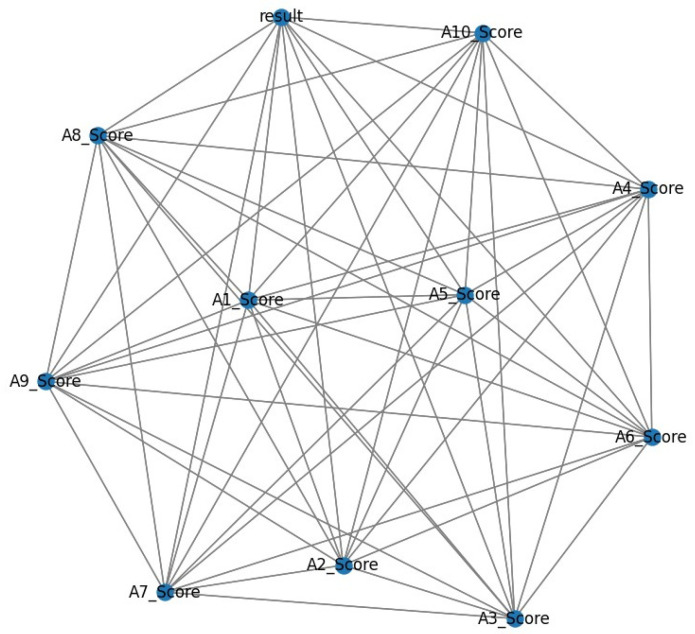
Network Graph of the Proposed Research.

Figure 6 represents the correlation heat map of the respective research. This map describes the similarities among the variables in the dataset. Here, each cell comprises of coefficient correlation. Additionally, a strong relationship is signified by 1, and 0 signifies neural relationships, whereas −1 signifies a relationship that is not strong in the system. From the figure, it is identified that there are better similarities in the variable. Accordingly, Figure 7 depicts the network graph of the proposed system. 

**Figure 8:**
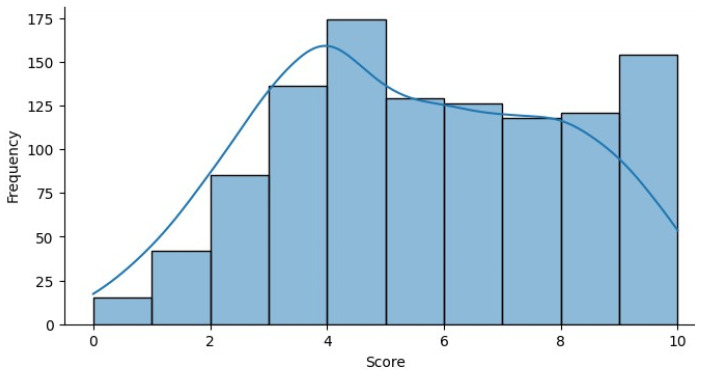
Q-Chat-10 Score Distribution.

Figure 7 represents the network graph of the respective graph. The network graph is the mathematical design to display the relation among the points in the dataset. Here, A1_score to A10_score points presented in the dataset and relation of the points are presented. The results in the figure are accomplished through the score of the ASD assessment with the Q-chat-10 dataset. Figure 8 explains the Q-chat-10 score distribution in the dataset. 

Figure 8 represents the score distribution of the Q-CHAT-10 dataset. The score signified in the graph represents the results of the ASD assessment in the dataset. The score is processed with the contributor’s answers for the 10 questions presented in the ASD assessment. Every bar in the histogram signifies the range of scores, which visualizes the distribution of scores in the Q-chat-10 Questionnaire. 

### 4.2. Performance Metrics

The performances of the respective mechanism are evaluated with certain performance metrics such as F1 score, accuracy, recall, and precision.

1. **Precision**

It is also denoted as identified positive figure and is given by the fraction of true positives to the average of true positives as well as false positives and given by Equation (12),

(12) $$Precision=\frac{Tp}{Tp+Fp}$$

2. **Recall**

It is given by the ratio of correctly identified results to overall identifications. The recall is also known as specificity or sensitivity and is given by Equation (13),

(13) $$Recall=\frac{Tp}{Tp+Fn}$$

3. **Accuracy**

It is defined as the ratio of correct identification to overall identification. The formula for accuracy is shown in Equation (14),

(14) $$Accuracy=\frac{Tp+Tn}{Tp+Fp+Tn+Fn}$$

4. **F1-score**

It is evaluated by the mean of recall and precision scores. It also denotes that if the F1-score predicted is higher, then the quality of the classifier is also high and given by Equation (15),

(15) $$F1-Score=2*\frac{Recall*Precision}{Recall+Precision}$$

The description of the Tp, Fp, Tn and Fn is illustrated in the following:

- Tp signifies the correct prediction of ASD in people with ASD
- Fp represents the incorrect prediction of ASD in people without ASD
- Tn describes the correct prediction of ASD in people without ASD
- Fn depicts the incorrect prediction of ASD in people with ASD.

### 4.3. Data Evaluation Results 

The section represents the outcomes of the dataset evaluation of the ASD dataset attained through the evaluation techniques such as chi-squared statistics and *p*-value. Table 8 represents the result of the dataset evaluation. 

Table 8 signifies the outcome of the dataset evaluation. The chi-square test is used to compare the observed results with expected results. Wherein the *p*-value is perceived to analyse the data. If the *p*-value is less or equal to 0.05, the result is trumpeted as significant, but if it is higher than 0.05, the result is non-significant and tends to be passed over. So, it is significant to interpret the chi-square test and *p*-value. From the results with the chi-squared statistic of 6.5735 and *p*-value of 0.0104, it is identified that there is a significant dependence among the variables that are involved in the chi-squared test. Moreover, *p*-value of 0.0104 is less than the chief level of 5%, which represents the better association among the variables. 

### 4.4. Experimental Results

**Figure 9:**
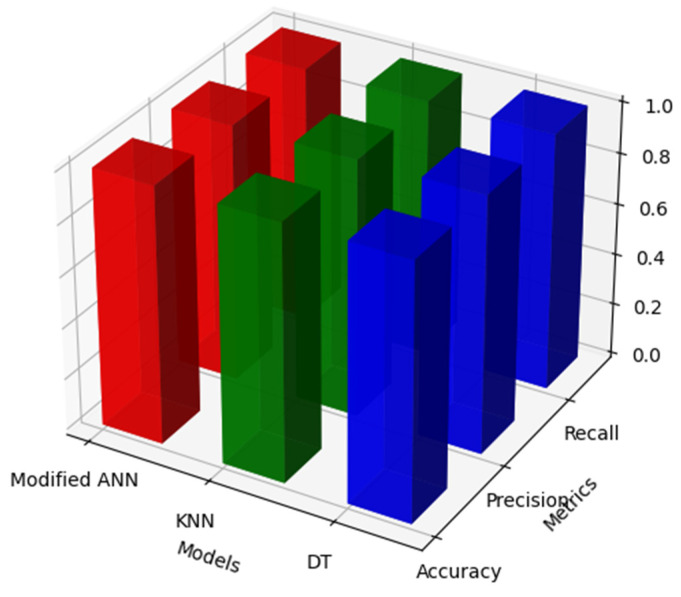
Signifies the Modified BAT with Modified ANN, KNN and DT Models.

The result accomplished by MBA optimisation with ANN, DT, and KNN classifiers is discussed in this section. The respective model is evaluated with a performance matrix such as F1 score, accuracy, recall, and precision. Table 9 and Figure 9 signifies the performance metrics of MBA with ANN, KNN, and DT classifier.

**Figure 10:**
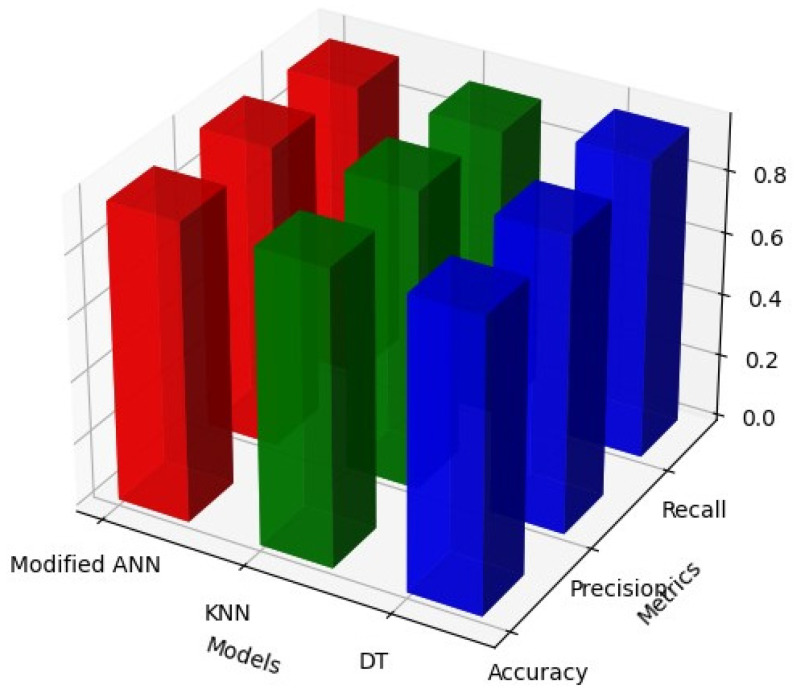
Outcome of BAT Algorithm with Modified ANN, KNN and DT Models.

Table 9 and Figure 9 signifies the performance metrics with MBA optimisation through modified ANN, KNN, and DT classifiers. From the results, it is revealed that the accuracy, precision, recall and F1-score of proposed modified ANN, KNN and DT classifiers is 1.00, 1.00, 1.00 and 1.00. Thus, the outcome attained with MBA optimisation through modified ANN, KNN and DT is significantly improved in the respective research. By utilising the MBA optimisation, all the classifiers attained better performance in ASD identification. Table 10 and Figure 10 signifies the performance metrics of traditional models KNN, ANN, and DT classifiers. 

Table 10 and Figure 10 signifies the performance of the BA optimisation through modified ANN, KNN, and DT classifiers. From the result, it is revealed that the accuracy, F1-score, precision, and recall of the ANN, KNN, and DT are 0.93, 0.85, and 0.93, which is less than the projected MBA optimisation results. Correspondingly, the outcome accomplished with BA optimisation through ANN is significantly low in the proposed system. 

### 4.5. Performance Analysis

**Figure 11:**
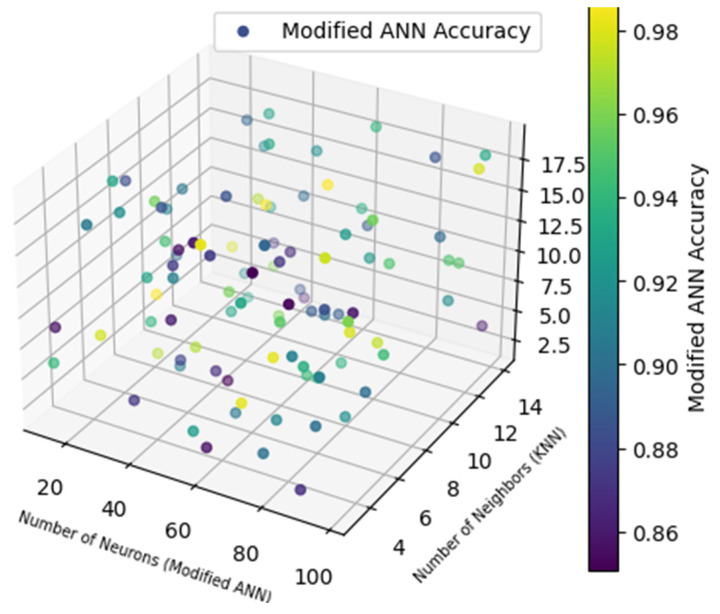
3D Visualisation of Metric of Modified ANN.

The projected ASD identification outcomes are analysed in this section. Here is the evaluation of BA optimisation with MBA optimisation in Classifiers KNN, ANN, and DT. Figure 11 signifies the 3D visualisation of the metrics for the Modified ANN. 

Figure 11 represents the 3D visualisation of the metric with the modified ANN on the basis of amount of neurons in modified ANN and amount of neighbours with KNN and DT. Here, it is identified that the neurons in modified ANN, neighbours in KNN and maximum depth of DT. Table 11 and Table 12 represents the results procured by Modified ANN and ANN models in the 10-fold test.

**Figure 12:**
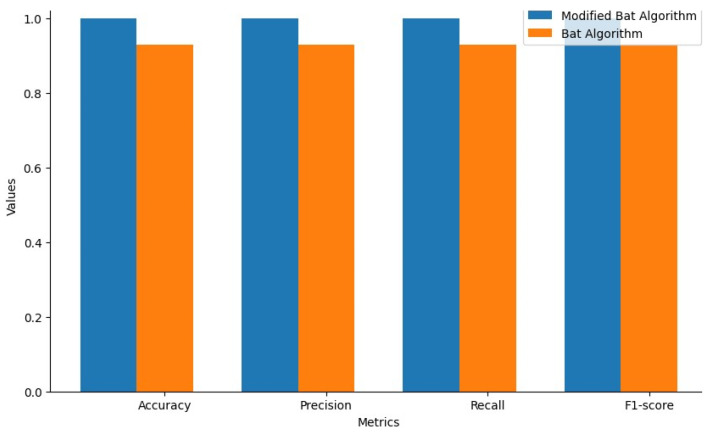
Performance Analysis of Modified BAT and BAT for Modified ANN System.

The study has also undergone 10-fold test for modified ANN and ANN, where data set are divided randomly into 10 different parts. Nearly, nine of those parts are for training and reserve one tenth for testing. This method is repeated ten times, each time reserving a different tenth for testing. When ten-fold cross validation approach is applied in modified ANN, the results are gradually increasing. From analysis, it is identified that the modified ANN acquired improved performance. Figure 12 signifies the performance analysis of the modified BAT and BAT algorithm for the modified ANN system. 

**Figure 13:**
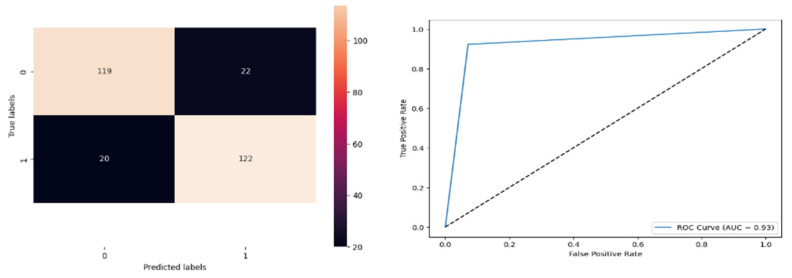
BA Optimisation in DT.

Figure 13 signifies the analysis of the efficiency of the Modified ANN with modified BAT and BAT algorithm. From the analysis, it is identified that the result of both the Modified BAT and BAT algorithm with the modified ANN is nearly similar in performance. Correspondingly, Figure 13 signifies the BA optimisation with DT.

**Figure 14:**
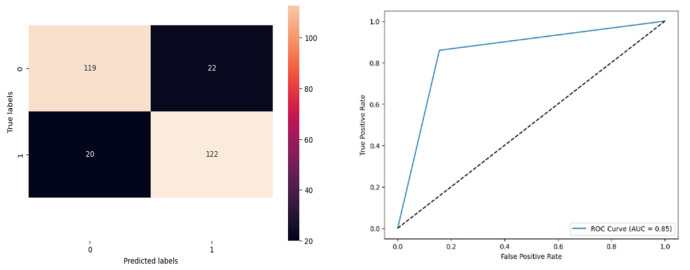
BA Optimisation in KNN.

Figure 13 denotes the accuracy, confusion matrix, and roc curve value in the BA optimisation in DT. It attains a roc curve value of 0.93. In the confusion matrix, the Tp, Fp, Fn, and Tn value are 119, 22, 20, and 122. Figure 14 signifies the BA optimisation in KNN.

**Figure 15:**
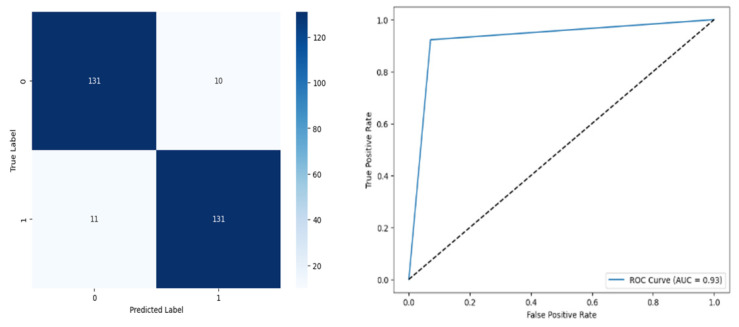
BA Optimisation in Modified ANN.

Figure 14 denotes the accuracy, confusion matrix, and roc curve value in the BA optimisation in KNN. It attains a roc curve value of 0.85. In the confusion matrix, the Tp, Fp, Fn, and Tn value are 119, 22, 20, and 122. Figure 15 signifies the BA optimisation in modified ANN.

**Figure 16:**
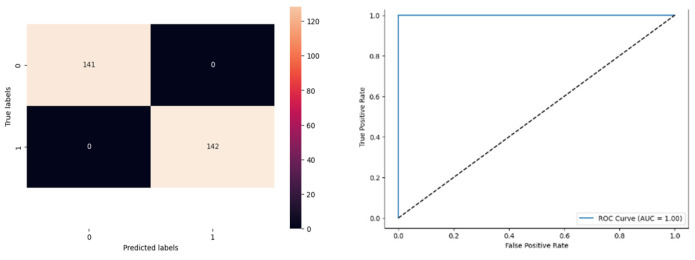
MBA Optimisation in DT.

Figure 15 denotes the accuracy, confusion matrix, and roc curve value in the BA optimisation in modified ANN. It attains a roc curve value of 0.93. In the confusion matrix, the Tp, Fp, Fn, and Tn value are 131, 10, 11, and 131. Figure 16 signifies the MBA optimisation in DT.

**Figure 17:**
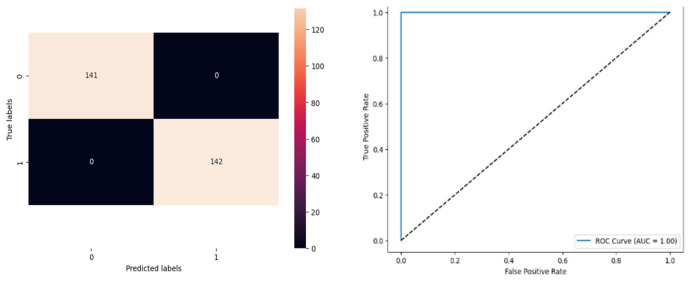
MBA Optimization in KNN.

Figure 16 denotes the accuracy, confusion matrix, and roc curve value in the BA optimisation in DT. It attains a roc curve value of 1.00. In the confusion matrix, the Tp, Fp, Fn, and Tn value are 141, 0, 0, and 142. Figure 17 signifies the BA optimisation in KNN.

**Figure 18:**
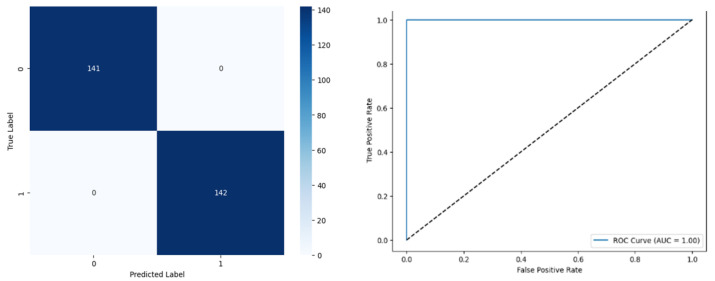
MBA Optimisation in Modified ANN.

Figure 17 denotes the accuracy, confusion matrix, and roc curve value in the BA optimisation in KNN. It attains a roc curve value of 1.00. In the confusion matrix, the Tp, Fp, Fn, and Tn value are 141, 0, 0, and 142. Figure 18 signifies the MBA optimisation in modified ANN.

Figure 18 denotes the accuracy, confusion matrix, and roc curve value in the BA optimisation in DT. It attains a roc curve value of 1.00. In the confusion matrix, the Tp, Fp, Fn, and Tn value are 141, 0, 0, and 142. 

The projected system utilised MBA optimisation with KNN, modified ANN, ANN, and DT classifiers to identify ASD through the Q-chat-10 dataset. The BA is utilised for the automated zooming capability for an effective finding of the optimal solution. Though it lacks convergence speed, accuracy, and fall for local extremum, the projected system utilised random perturbation of trend and optimal orientation to improve the system’s efficacy. From the experimental outcome, the projected system with MBA attained an accuracy 1.00 in KNN, ANN, and DT, whereas the BA optimisation in classifiers attained an accuracy of KNN OF 0.85, modified ANN of 0.93, and DT of 0.93. The proposed identification model is examined with certain metrics to calculate the performance of ASD identification. Considering the capability of BA, the projected model utilised MBA in ANN, KNN, and DT classifiers to enhance the identification with better convergence speed and accuracy. Therefore, the MBA with ANN, KNN, and DT model on the Q-chat-10 dataset attained better results which are verified over the results.

### 4.6. Insights and Discussions

The involvement of the respective classification system and the utilised dataset is illustrated in the section. Generally, conventional researches concentrated on the autism detection on all set of people [26,27,32]. The distinct attention on the autism on aiming children and adolescence is limited. The respective research fill this gap by utilising the Q-chat-10 dataset. Moreover, the classical system used the particular symptoms of the autism such as eye movement features for the classification of autism [40,45,46,47,48]. The utilised children dataset comprises the assessment answers, in which all the behavioural symptoms are covered in the examination. Furthermore, images are used to identify the ASD in some exiting models [44]. In the case of ASD, it is necessary to analyse all the behavioural symptoms. For that, an effective assessment is needed to cover most of the symptoms. To attain effective classification of the children- and adolescence-related autism, respective research used the assessment based dataset. The uses and functions of the dataset are illustrated in Table 13. 

Subsequently, several datasets utilised in the conventional ASD identification lack in enhancing the efficiency, specificity, predictive, and sensitivity in the screening of ASD. Accordingly, accuracy is the significant metric used to calculate the performance of the model. Several conventional methods lacked in the important metric accuracy [38,44]. The proposed model uplifted the performance using MBA optimisation with KNN, modified ANN, ANN, and DT classifiers and accomplished higher accuracy of 1.00 with modified ANN, 1.00 with KNN and 1.00 with DT. The outcomes reveal the effective performance of the proposed method. Due to the lack of utilised dataset in the existing methods, a respective model is compared with the classical algorithms such as KNN, ANN, modified ANN and DT. From the comparative analysis result, the proposed model outperformed all the three traditional algorithms by attaining 0.7 and 0.15 higher than the classical algorithm. Correspondingly, the proposed research is anticipated to contribute in the research related to autism and to support the physicians in the diagnosis of the autism to enhance the quality of life of children and adolescents with ASD. 

## 5. Conclusions

ASD is a lifetime behavioural disorder commonly caused by genetic, social, and environmental factors. It impacts the social life of ASD patients by affecting the capability of social skills like communication, interaction, learning, etc. Quick identification was needed for early treatment to improve the standard of ASD patient’s life. The traditional screening was a time-consuming and expensive process that included behavioural assessment with the help of qualified doctors. Enormous conventional methods achieved satisfactory results in the identification system but lacked in handling large data sets, accuracy, and speed. Therefore, the projected identification system employed MBA optimisation in ANN, modified ANN, DT, and KNN classifiers to enhance the efficacy of ASD identification. This system utilised BA because of its automatic zooming capability for effectively finding optimal solutions. However, it lacks certain drawbacks, like falling into local extremum, accuracy of optimization, and speed of convergence. To improve the performance of BA, the projected model utilised random perturbation of trends and optimal orientation. The features of the projected model utilised the Q-chat-10 dataset, which was acquired from the assessment through the questionnaire comprising 10 questions taken by the contributing children’s parents. It comprises four age groups: toddlers, children, adolescents, and adults. To examine the excellence of the dataset, dataset evaluation such as chi-squared statistic and *p*-value are utilised in the proposed model. The outcome of the chi-squared statistics test is 6.5735 and *p*-value is 0.0104, which represents better correlation among the variables in dataset. Further, the performance of the projected identification system was calculated with certain metrics. The outcome revealed that the projected model attained accuracy, F1-score, recall, and precision of 1.00, 1.00, 1.00, and 1.00 in modified ANN classifier ensuring improved performance when compared with other state-of-the-art methods. The projected identification system was intended to assist physicians and researchers in enhancing the diagnosis of ASD to improve the standard of life for ASD patients. In future, the proposed method can be further accessed in different other disease detection and diagnosis with improved efficiency. In addition, the severity of ASD can also be identified by using the proposed approach.

## Figures and Tables

**Table 1:** Regions of the Brain and their Functions Affected by ASD.

S. No	Region of Brain	Functions
1.	Hippocampus	It is the area of the brain responsible for the storing memories
2.	Amygdala	It is responsible for emotional response. The size of the amygdala differ among people with ASD and without ASD. People with ASD have a smaller size of amygdala than people without ASD, or it becomes smaller in the case of anxiety
3.	Cerebellum	It regulates motor activity and body movements. People with ASD have a decreased number of brain tissue in the cerebellum
4.	Cerebral Cortex	It is responsible for behavioural reactions. The outer layer of the brain have different thickness patterns in people with ASD and without ASD.
5.	Brain Stem	It regulates enormous body functions
6.	Basal Ganglia	It regulates emotions, behaviours, executive functions, and motor learning
7.	Corpus Callosum	It ensures that both sides of the brain are in communication and sending signals

**Table 2:** Attributes of the Dataset.

Characters	Kind	Specification
Contributor’s age	Numbers	Years
Contributor’s gender	String	Female or Male-
Contributor’s ethnicity	Text format-String	Common type of ethnicity
Contributor’s family with PDD	Boolean	Yes or No
Contributor’s health history with Jaundice from birth	Boolean	Yes or No
Contributor’s type	String	Guardian, Self, Parent, staff of medical, doctor, etc.
Contributor’s Residential Country	String	Countries-Text format
Prior use of screening app details	Boolean	Yes or No
Type of identification-AGE_DESC	Value in integer-0, 1, 2, 3	Toddler-0, children-1, adolescent-2 and adult-3

**Table 3:** Question in ASD Screening for Q-chat-10 Dataset.

S. No	Assessment Questions in the ASD Screening for Children Dataset
1	Is the kid giving unconnected responses to queries?
2	Is the kid not replying to their own name?
3	Is the kid not involved in pretend sports with others?
4	Is the kid slow to recognizing the feelings of others?
5	Is the kid distressed by changes, even if it is small?
6	Is the kid having interests which are compulsive?
7	Is the kid sensitive to touch, taste and smells?
8	Is the kid hesitant to mingling with others?
9	Is the kid avoiding physical connection?
10	Is the kid showing slight awareness of threat circumstances?

**Table 4:** Modal Summary of ANN Algorithm.

ANN	Hidden Layers	Neurons per Hidden Layer	Activation Function	Optimizer	Learning Rate	Epochs
ANN	2	15	ReLU	Adam	0.001	100

**Table 5:** Hyperparameters of Modified ANN Algorithm.

Hyperparameter	Value
Number of Hidden Layers	2
Neurons in the First Hidden Layer	50
Neurons in the Second Hidden Layer	15
Activation Function	ReLU
Learning Rate	0.001
Optimizer	Adam
Batch Size	64
Number of Epochs	100
Weight Optimization	V-Weight Optimization Based ANN

**Table 6:** Hyperparameters of DT Algorithm.

Hyperparameter	Value
Number of Neighbours	5
Distance Metric	Euclidean

**Table 7:** Hyperparameters of KNN Algorithm.

Hyperparameters	Value
Criterion	Gini
Max Depth	10
Min Samples Split	2
Min Samples Leaf	1

**Table 8:** Result of the Dataset Evaluation.

S. No	Methods	Outcomes
1	Chi-squared Statistics	6.5735
2	*p*-Value	0.0104

**Table 9:** Performance Metrics of MBA.

Model	Accuracy	Precision	Recall	F1-Score
Modified ANN	1.00	1.00	1.00	1.00
KNN	1.00	1.00	1.00	1.00
DT	1.00	1.00	1.00	1.00

**Table 10:** Performance Metrics of Traditional Approaches.

Model	Accuracy	Precision	Recall	F1-Score
ANN	0.93	0.93	0.93	0.93
KNN	0.85	0.85	0.85	0.85
DT	0.93	0.93	0.93	0.93

**Table 11:** 10-fold test for Modified ANN.

Fold	Accuracy	Precision	Recall	F1 Score
1	0.85	0.88	0.82	0.85
2	0.82	0.86	0.79	0.81
3	0.88	0.91	0.87	0.89
4	0.89	0.92	0.88	0.9
5	0.86	0.89	0.84	0.87
6	0.9	0.93	0.89	0.91
7	0.9	0.9	0.9	0.9
8	0.91	0.94	0.9	0.92
9	1	1	0.98	0.98
10	1	1	1	1

**Table 12:** 10-fold test for ANN.

Fold	Accuracy	Precision	Recall	F1 Score
1	0.65	0.65	0.6	0.65
2	0.67	0.6	0.67	0.67
3	0.72	0.72	0.72	0.72
4	0.75	0.75	0.7	0.75
5	0.771	0.7	0.7	0.77
6	0.79	0.79	0.79	0.79
7	0.81	0.81	0.81	0.81
8	0.825	0.825	0.825	0.825
9	0.83	0.83	0.83	0.83
10	0.83	0.83	0.83	0.83

**Table 13:** Involvement of the Dataset in ASD Screening.

ASD Assessment in Q-Chat-10 Dataset
**Uses**
It is utilised in the ML systems for classifying ASD. It is used to enhance treatments for ASD. It is used to improve the life standard of people with ASD and their loved ones.
**Functions**
It is used to access the prevalence of ASD. It is used to make advancements in screening tools for ASD. It is used to enhance the diagnosis of ASD.

## Data Availability

Data are contained within the article.
